# Breast Cancer Bone Metastasis: A Narrative Review of Emerging Targeted Drug Delivery Systems

**DOI:** 10.3390/cells11030388

**Published:** 2022-01-24

**Authors:** Huimin Shao, Pegah Varamini

**Affiliations:** 1 Faculty of Medicine and Health, School of Pharmacy, The University of Sydney, Sydney, NSW 2006, Australia; hsha0542@uni.sydney.edu.au; 2 Sydney Nano Institute, The University of Sydney, Sydney, NSW 2006, Australia

**Keywords:** breast cancer, bone metastasis, targeted drug delivery system, nanomedicine, nanotechnology

## Abstract

Bone is one of the most common metastatic sites among breast cancer (BC) patients. Once bone metastasis is developed, patients’ survival and quality of life will be significantly declined. At present, there are limited therapeutic options for BC patients with bone metastasis. Different nanotechnology-based delivery systems have been developed aiming to specifically deliver the therapeutic agents to the bone. The conjugation of targeting agents to nanoparticles can enhance the selective delivery of various payloads to the metastatic bone lesion. The current review highlights promising and emerging advanced nanotechnologies designed for targeted delivery of anticancer therapeutics, contrast agents, photodynamic and photothermal materials to the bone to achieve the goal of treatment, diagnosis, and prevention of BC bone metastasis. A better understanding of various properties of these new therapeutic approaches may open up new landscapes in medicine towards improving the quality of life and overall survival of BC patients who experience bone metastasis.

## 1. Introduction

Breast cancer (BC) is the most common malignant tumour among women [1]. According to the American Cancer Society (ACS) reports, BC leads to the second-highest cancer-related deaths in women after lung cancer. One in 38 women (about 2.6%) will die from BC. There were 2.3 million new cases in 2020 that led to 685,000 deaths globally [2]. In addition, the ACS pointed out that BC’s incidence rate increased about 0.3% per year in recent years [3]. The mortality rate also increased significantly from 1990–2015 [4]. Based on the immunohistochemical expression of estrogen receptor (ER), progesterone receptor (PR), and human epidermal growth factor receptor 2 (HER2), BC can be classified into four major subtypes: hormone receptor (HR)+/HER2+, HR+/HER2−, HR−/HER2+ and HR-/HER2− [5,6]. In the absence of all three receptors, i.e., HR−/HER2− subtype, triple-negative breast cancer (TNBC), accounts for 15–20% of all BCs [7].

According to the molecular profiles, BC could be classified as luminal subtype, HER2 enriched+ subtype, and basal-like subtype with a high expression of basal markers [8]. The luminal subtype could be divided into luminal A and luminal B tumours. The luminal A that comprises 40% of all subtypes shows the best clinical prognosis with a high level of ER expression. Therefore, these patients are more likely to benefit from hormonal therapy alone. The other less common subtype, luminal B (20%) tumours, express ERs at a lower level but exhibit higher levels of proliferation-related genes. Consequently, patients within this category may need chemotherapy [9]. HER2 enriched tumours (15%) also show overexpression of proliferation-related genes. HER2+ tumours that are ER- are classified as luminal B subtype [10]. The basal-like group is characterised by the upregulation of genes expressed by basal/myoepithelial cells. Although it has been reported that 71% of TNBC tumours were found to be basal-like and 77% basal-like tumours were triple negative, TNBC and basal-like BC are not synonyms [8]. TNBC can be further divided into six subtypes: basal-like (BL1 and BL2), mesenchymal (M), mesenchymal stem-like (MSL), immunomodulatory (IM), and luminal androgen receptor (LAR), and an unspecified group (UNS) [11].

Metastasis is one of the main reasons for the high mortality rate among BC patients. About 20–30% of recurrences among early BC patients are accompanied by metastatic diseases [12]. Common metastatic sites for breast cancer are bones, liver, lungs, and brain [13]. According to recent research, bone is the most common metastatic organ, while the brain is the least. In addition, different subtypes of BCs have variable likelihoods of developing metastasis. For example, the HER+ BC and TNBC are more aggressive and more likely to develop metastasis [6]. Some studies indicate that bone metastases are more frequent in HR+ subtypes than all other BC subtypes [14,15]. Bone metastasis influences the quality of patients’ life by inducing skeletal-related events (SREs), such as bone pain and tumour-induced fracture, and decreases survival [16]. The average five-year survival rates for the patients with local-regional and metastatic recurrence are 80% and 25%, respectively [17].

## 2. Mechanism of BC Bone Metastasis

The BC metastasis process includes several complex steps: invasion, migration, adhesion, and survival of the tumour cells at the metastatic sites. The ‘‘seed and soil’’ theory was first reported in 1889. This means the development of cancer metastasis depends on the interactions between the ‘‘seeds’’, which indicate the tumour cells, and the ‘‘soil’’, which suggests the microenvironment of the potential metastatic site [18].

### 2.1. Invasion

In metastasis development, BC cells at the primary tumour site first induce angiogenesis to ensure they have access to nutrients for proliferation. The newly developed blood vessels also provide a pathway for the tumour cells to enter the blood and lymphatic vessels [19]. Like normal epithelial tissue cells, there are multiple tight cell connections between BC cancer cells, which is not conducive to cell movement and invasion. Thus, the BC cells need to lose some epithelial phenotype by going through epithelial-to-mesenchymal transition (EMT) [20]. Then, at the primary tumour site, the BC cells induce the secretion of a major class of matrix metalloproteinase (MMPs) essential for tumour cell invasion. Under the action of MMPs, the tumour cells break through the basement membrane and enter the extracellular matrix and surrounding normal tissues [21].

### 2.2. Migration

After the invasion, the tumour cells enter the blood circulation and become circulating tumour cells (CTCs), disseminating to the bone via blood vessels or lymphatic vessels. The CTCs may travel as single cells or as multicellular clumps. These clumps can persist in the circulation system until encountering small-calibre capillaries. Most of the CTCs may be cleared immediately. However, clusters of CTCs can move through microvessels by holding together their adhesive interactions to form a single-cell chain [22]. Finally, the CTCs arrive at the bone and enter the parenchymal tissue by degrading the vascular basement membrane and penetrating the blood vessel. In the bone microenvironment, the CTCs eventually become disseminated tumour cells (DTCs). To adjust the bone microenvironment, DTCs change their biological phenotype. For example, DTCs can avoid destruction and last a long time in the tissue parenchyma [23]. Additionally, DTCs may survive by their capability of withstanding anoikis, for instance, through expressing the tyrosine kinase receptor TrkB [24] or through non-canonical WNT signalling [25].

Additionally, the bone induces the expression of several cytokines and chemokines, which can further promote the BC cells’ homing and attract more primary BC cells. It has been proven that the behaviour of C-X-C motif chemokine receptor type 4 (CXCR4 and CXCL12, receptor activator of NF-kB (RANK) and RANK ligand (RANKL), are of great importance to the homing of BC cells to the bone. CXCL12 is a chemokine derived by the osteoblast related to homing of osteogenic precursors to bone marrow [26]. BC cells overexpressing CXCR4 are more likely to be recruited by bone-derived CXCL12 [27].

Furthermore, the RANKL/RANK/osteoprotegerin (OPG) system plays a vital role in regulating bone formation and resorption. RANKL could induce bone resorption by binding to the RANK located on the osteoclast surface, while OPG secreted by osteoblast prevents RANKL from binding to RANK by acting as a decoy receptor for RANKL. The ratio of OPG to RANKL indicates the bone remodelling, of which the high ratio means bone formation while the low ratio represents bone resorption [28]. It has been proven that RANKL could induce bone metastasis by recruiting RANK-expressing cells directly to bone [29].

### 2.3. Adhesion

DTCs adhere to the favourable metastatic microenvironment, usually bone marrow for bone metastasis [30]. This step is mainly mediated by integrin and cadherin. Integrin is an isodiglycan protein that belongs to a heterodimeric transmembrane glycoprotein family. Integrins can mediate the BC cells’ adhesion to the extracellular matrix. Among the whole family, the ανβ3 integrin plays a vital role in regulating the migration of BC cells to the trabecular bone by binding to a tripeptide (Arg-Gly-Asp) present in vitro nectin, osteosialin, and osteopontin [31,32,33,34].

E-cadherin is an epithelial-specific transmembrane glycoprotein mediating cell-cell adhesion, maintaining normal cells’ cellular polarity and epithelial morphology. This glycoprotein plays an essential role in maintaining epithelial phenotype, and the loss of E-cadherin may give tumour cells the properties of migration and invasiveness [35,36,37,38,39]. Furthermore, E-cadherin and N-cadherin mediate the interaction between BC cells and bone marrow stromal cells, promoting the homing progress of BC cells [40]. Cadherin-11 has been proven to be overexpressed in various favourable metastatic sites for BC, such as brain, lung, and importantly, bone. The migrative and invasive capability of BC cells will be reduced when cadherin-11 is inhibited, indicating the vital role it plays in the process of BC bone metastasis [41].

### 2.4. Survival

**Figure 1:**
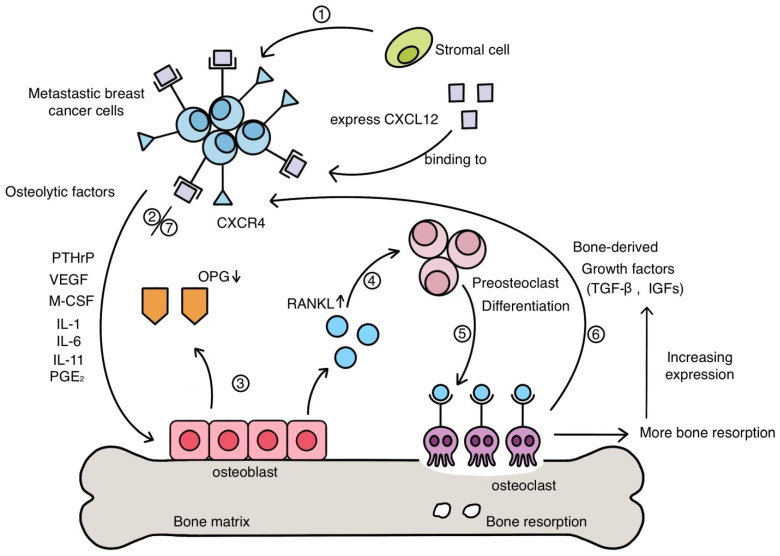
An overview of the “vicious cycle of bone metastasis’’ (the figure is reproduced from reference [44] with modifications to the original source). A vicious cycle happens when tumour cells secrete osteoclast-stimulating factors, while bone marrow stromal cells secrete tumour growth factors.

After adhesion to the bone tissue, the proliferation of BC cells in the bone is the last but vital step of metastasis. The survival of tumour cells relies on the ‘‘vicious cycle of bone metastasis’’ (Figure 1) [42]. In the bone micro-environment, BC cells firstly overexpress parathyroid hormone-related protein (PTHrP), which subsequently stimulates the expression of RANKL and suppresses the expression of OPG [42]. When the balance between OPG and RANKL is broken, the function of osteoclasts is promoted, which results in bone resorption. During this process, the release of growth factors, such as transforming growth factor-β (TGF-β), vascular endothelial growth factor (VEGF), insulin-like growth factors (IGFs), BMPs, and calcium, is ongoing in the bone microenvironment. It was proven that the increase in intracellular calcium levels among BC cells could interfere with the osteogenic function and facilitate survival [43].

**Figure 2:**
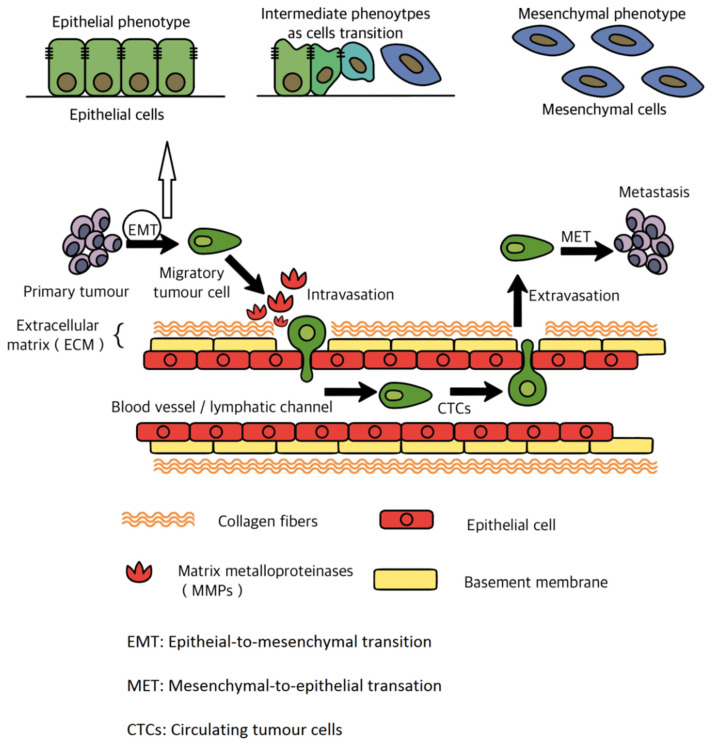
An overview of invasion and metastasis (the figure is reproduced from reference [50] with modifications to the original source).

TGF-β is a pleiotropically expressed multifunctional cytokine that controls tissue homeostasis by regulating cellular apoptosis, proliferation, and differentiation [45]. In metastasis progression, the TGF-β signal can suppress E-cadherin expression by activating the TGF-β receptors and phosphorylating the receptor-activated SMAD2 and SMAD3 [46]. In addition, TGF-β can upregulate the expression of pre-osteolytic factors such as PTHrP [47]. It has been proven that BC cells in metastatic bone sites express larger quantities of PTHrP compared to the cells in the primary breast site [48]. These factors enhance BC cell proliferation and remodelling the bone microenvironment by inducing angiogenesis and osteoclastogenesis, making the bone microenvironment more suitable for BC cells to colonise. TGF-β, IGFs, BMPs as well as the high level of calcium can simultaneously stimulate the overexpression of PTHrP and those growth factors mentioned above, leading to osteoclastic bone destruction. Subsequently, the resorbed bone further promotes the expression of TGF-β and IGFs. The release of these factors increases the amount of PTHrP produced by BC cells. As a result, more bone resorption is caused, which eventually promotes a vicious cycle of bone metastasis [49]. With the help of the appropriate bone microenvironment, BC cells can proliferate in the bone. An overview of invasion and metastasis is illustrated in Figure 2.

## 3. Conventional Treatments of BC Bone Metastasis

The main therapeutic aims of metastatic BC treatment are to prolong life and relieve symptoms [51]. In the past few decades, the primary therapeutic methods for BC bone metastasis have been divided into systematic and local treatments. The former includes systemic administration of anti-resorptive, antitumour compounds, and radiopharmaceuticals, while the latter includes surgery and radiation therapy. The key challenge is that most antitumour agents are cytotoxic, leading to severe nephrotoxicity, hepatotoxicity, and adverse effects on other normal organs if not selectively delivered to metastatic lesions. Moreover, the administration of anti-resorptive compounds may exert negative impacts on healthy bone tissue. Although local palliative treatments could help extend the patients’ overall lifespan, they cannot significantly improve the survival rate. Furthermore, the severe side effects during the treatment seriously impact the patients’ quality of life [51,52,53,54]. All these facts indicate an urgent need for targeted therapy for BC bone metastasis to increase survival and, at the same time, reduce the adverse effects. Conventional treatments of BC bone metastasis are listed in Table 1.

## 4. Targeting Agents for BC Bone Metastasis

**Figure 3:**
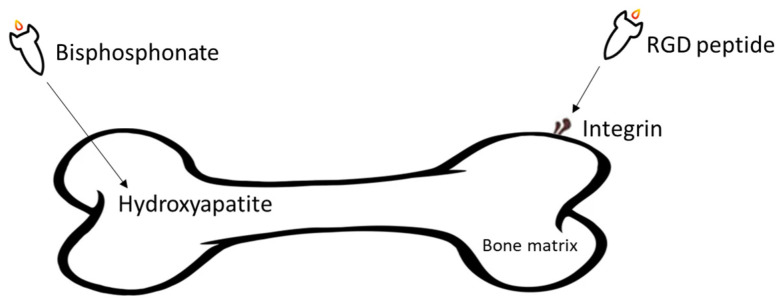
Overall illustration of targeting agents utilised in the development of DDSs aiming at treating BC bone metastasis.

In targeted DDSs, the targeting agents play vital roles. The drug can be accurately delivered to the metastatic tumour sites with targeting agents, increasing the treatment efficacy and decreasing side effects generated by some cytotoxic compounds. Various bone targeting agents include tetracycline, bisphosphonate, γ-carboxylated glutamic acids (Gla) and some amino acids (e.g., aspartic acid (Asp), and glutamic acid (Glu)), and aptamers are investigated in different cancers [56]. However, the most commonly used bone targeting agents for DDSs have involved Arginine-Glycine-Aspartic acid (RGD) peptide [57], and two drugs from bisphosphonates family, alendronate [58] and zoledronic acid [59] (Figure 3). These agents have been found to have high potential to be employed in the development of bone targeted pharmaceuticals.

### 4.1. Arginine-Glycine-Aspartic Acid (RGD) Peptide and Its Derivative

Integrins are a group of divalent cation-dependent heterodimeric membrane glycoproteins composed of α and β subunits, playing vital roles in cell-cell and cell-extracellular matrix (ECM) adhesion [60]. Among all subtypes of integrins, overexpression of ανβ3 integrin has been proven to be related to BC bone metastasis and poor prognosis and decreased survival time of BC patients [61]. As an integrin predominantly expressed in blood vessels, ανβ3 integrin can mediate angiogenesis, cell proliferation, and metastasis in several types of cancers. If ανβ3 integrin is blocked with integrin antagonists, angiogenesis of some tumour cells, such as melanoma, prostate cancer, and BC cells, would be disrupted [62,63]. Furthermore, by binding to fibronectin, fibrinogen, or osteopontin, ανβ3 integrin induces the migration of endothelial cells, and it activates several signalling cascades, which protect the cells from apoptosis [62,64].

RGD is an arginine-glycine-aspartic acid (Arg-Gly-Asp) tripeptide, which can bind specifically to ανβ3 integrin. Several preclinical studies showed that RGD peptide successfully blocked osteoclast-mediated osteolysis in bone metastatic animal models by acting as ανβ3 integrin antagonist [65]. As a peptide selectively binding to ανβ3 integrin, RGD peptide can be conjugated on drug delivery systems (DDSs) for targeted tumour therapy. Recently, RGD peptides conjugated DDSs have been widely studied in prostate cancer and bone metastasis [66,67,68]. Thus, targeting ανβ3 integrin with RGD peptide provides a promising way to treat BC metastasis.

### 4.2. Bisphosphonate

**Figure 4:**
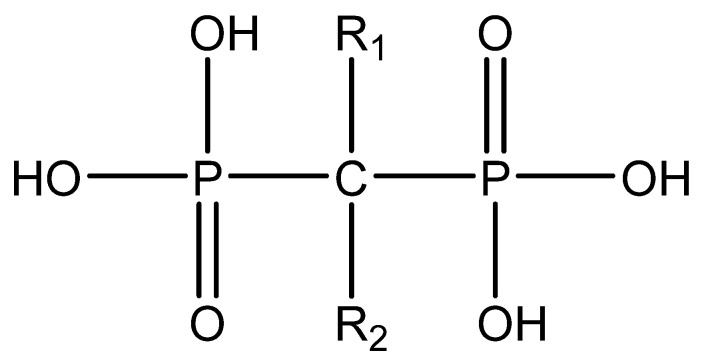
The general molecular structure of bisphosphonates.

Bisphosphonates (BPs) are a group of chemical compounds showing high affinity to hydroxyapatite (HA) crystals commonly seen in bones and teeth. The reason for this high affinity is that BPs can generate bidentate or tridentate chelation with the calcium ion on the HA [69]. The BPs can recognise and localise quickly to tissues where HA is present after intravenous or oral administration. Thus, conjugation of BPs to the DDSs provides a promising strategy to target specifically to the bone [70]. Furthermore, BPs are widely used in the treatment of conditions where bone resorption occurs. BPs can be selectively taken up by osteoclasts [71]. By inactivating osteoclasts, they can simultaneously exert specific auxiliary therapeutic effects on SREs, such as increasing bone density, decreasing fracture risk, and relieving bone pain at the metastatic sites while playing the targeting role [72]. Different BPs can be distinguished by side-chain groups at R1 and R2 sites (Figure 4).

Various functional groups at R1 and R2 can affect the ability of BP to bind to HA and their antiresorptive efficacy, respectively. For example, with a hydroxyl group at the R1 side chain, the binding capacity of BPs increases significantly compared to those having a hydrogen (tiludronate) or chlorine (clodronate) on this carbon. The reason is that they can form a tridentate instead of bidentate chelation between BPs and calcium ions [73]. The antiresorptive capability of alendronate, neridronate, risedronate, olpadronate, ibandronate, zoledronic acid, and pamidronate is 10–10,000 times stronger than non-nitrogenous BPs (tiludronate, clodronate, and etidronate). This is mainly due to the presence of nitrogenous functional groups on the R2 side chain [74,75]. The results from kinetic studies of the HA crystal growth showed that the ranking of the capability of binding to HA at neat surfaces among BPs is zoledronic acid > pamidronate > alendronate > ibandronate > risedronate > etidronate > clodronate [76]. BPs from different generations and their structural differences are illustrated in Table 2.

Besides, it has been reported that BPs could have an antitumour effect directly and indirectly among several tumours, such as prostate, lung, and melanoma cancer cells, in vitro. The potential mechanism of these antitumour activities is that BPs could mediate apoptosis and the cell cycle arrest, which suggests synergistic therapeutic effects with anticancer agents [77]. Among all BP analogues, alendronate and zoledronic acid are most frequently used as targeting agents in nanoparticles.

#### 4.2.1. Alendronate

Alendronate, a second-generation BP, has been used as a bone-targeting agent in treating metastatic lung cancer [78], breast cancer [79], primary bone cancer, and metastatic bone cancer [80]. More importantly, a study by Rouach et al. showed that in early breast cancer in postmenopausal patients, a previous history of oral alendronate consumption is linked with a lower likelihood of bone metastases [79]. In addition, alendronate has been extensively used for functionalising nanoparticles to achieve various targeted delivery to the bone.

#### 4.2.2. Zoledronic Acid

Zoledronic acid, a third-generation BP, has been proven to be a cost-effective solution in treating bone resorptive diseases, such as osteoporosis and osteoporotic fractures [81]. The administration of zoledronic acid could significantly reduce the pain and improve the quality of life among BC patients with bone metastasis in the clinic [82], suggesting an additive therapeutic effect. Due to a high affinity to bone, zoledronic acid has strong targeting potency and can be employed as a targeting agent in DDSs to treat BC bone metastasis.

## 5. Advanced Targeted DDSs

Nanoparticles have been under investigation for a few decades because of their capability to alter the drug’s pharmacokinetics. The introduction of nanoparticles can solve the poor solubility of some hydrophobic drugs and reduce the metabolism, thus preventing the drug compounds from being degraded in the microenvironment [83,84]. The utilisation of nanoparticles for effective delivery of active pharmaceuticals could also promote the enhanced permeability and retention effect (EPR), which is widely observed in the vasculature of tissues undergoing pathologies [85]. Notably, only nanoparticles whose sizes are no more than 200 nm have the property of easily penetrating through mucus without being removed by the natural size-filtering mechanism [86].

The surface of the nanoparticles can be modified as needed to achieve specific requirements of different disease conditions [87]. Decorated with different targeting agents, the efficient delivery of various functional agents could be realised by nanoparticles. The most common targets for BC bone metastasis are HA and ανβ3 integrin. Alendronate [88] and zoledronic acid [59] are the most commonly used HA targeting agents among all BPs. The successful targeting of ανβ3 integrin is realised by involving RGD peptide into the DDSs [66,67,68]. To achieve the goal of treatment, diagnosis, and prevention of BC bone metastasis, various classes of compounds, including anticancer therapeutics [78,89], contrast agents [90], photodynamic [91], and photothermal materials [59] have been used to deliver to the bone. The following section discusses these emerging advanced DDSs depending on the nature of payloads used as described above.

### 5.1. Targeted DDSs Loaded with Anticancer Agents

#### 5.1.1. Cisplatin Prodrug

To deliver cisplatin prodrug (DSP) to BC bone metastatic lesions, He and co-workers developed Zn^2+^ coordination polymer particles coated with alendronate-conjugated polyethylene glycol (PEG). The nanoparticle (DSP-Zn@PEG-ALN NP) had an average size of about 55 nm, which endowed the nanoparticles the ability to penetrate the slits (80 nm) of the bone sinusoidal capillaries and successfully transfer to the metastasis. Results from the in vitro HA binding test suggested that targeted DSP-Zn@PEG-ALN NPs had higher binding potency to the bone than non-targeted nanoparticles and free cisplatin [92]. In in vivo biodistribution assay, DSP-Zn@PEG-ALN NPs showed the most increased localisation in the bone after tail vein administration among MDA-MB-231 bearing mice. Furthermore, the platinum concentration in metastatic sites was up to 4-fold higher than that of healthy bone.

Moreover, the administration of DSP-Zn@PEG-ALN NPs could suppress the growth of tumour and decrease bone destruction in a mouse BC bone metastasis model induced by intra-tibia injection of MDA-MB-231 cells. Importantly, researchers assessed the analgesic effects of DSP-Zn@PEG-ALN NPs in the mice group treated with these NPs. These mice showed significantly less duration of paw lifting and number of flinches, which suggested that the targeted NPs could effectively reduce the pain caused by metastasis to bone and potentially improve the quality of life. Moreover, the in vitro release assay result indicated that the release of DSP-Zn@PEG-ALN NPs was promoted as the pH reduced compared to the physiological pH (pH = 7.4). Thus, combining the pH-sensitive properties and specific release of NPs in the acidity of the bone microenvironment and the bone targeting capability, the utilisation of DSP-Zn@PEG-ALN NPs could prevent other organs from cisplatin toxicity [92].

Huang and co-workers reported an alendronate-modified nanoparticle (DZ@ALN) loaded with a cisplatin prodrug and zoledronate. Apart from high affinity for the bone, DZ@ALN could also mediate the activation of the osteoclasts both in vitro and in vivo. Furthermore, compared to the combination of free cisplatin prodrug and zoledronate, DZ@ALN could significantly inhibit tumour growth in vivo in a bone metastasis tumour model induced by intra-tibia injection of MDA-MB-231 cells, relieve bone pain, and prevent bone destruction, which further breaks the ‘‘vicious cycle’’ of BC bone metastasis [93].

#### 5.1.2. Bortezomib

Bortezomib is a protease inhibitor widely investigated in treating various cancers, such as myeloma, prostate, and lung cancers. Wang and co-workers developed a tripeptide RGD-targeted dendrimer for the delivery of bortezomib to metastatic tumour sites. With a boronate-catechol linkage, the dendrimer was endowed with pH-responsive properties. The result from in vitro targeting assay indicated that the conjugation of RGD could successfully lead the dendrimers to the MDA-MB-231 cells. Besides, the X-ray and micro-CT results showed that the bone destruction of the tibias in the RGD-targeted nanoparticle treated group was the lowest while the bone volume and trabecular number were the highest compared to the control groups. These results suggested that the RGD-targeted dendrimer could suppress the osteolysis caused by BC cells [57].

Zhu and co-workers developed alendronate-modified bortezomib-catechol loaded prodrug micelles. The bortezomib-catechol-loaded ALN-NPs showed better release in an acidic environment (pH = 5.0) compared to the physiological pH. They also observed an enhanced affinity to the bone compared to the control groups, which could result in reduced systemic toxicity. In vivo, the drug-loaded alendronate-NPs could significantly repress tumour growth and tumour-induced bone destructions among female MDA-MB-231 cell bearing BALB/c-nu mice [94].

#### 5.1.3. Curcumin

Redox-sensitive alendronate targeting micelles loaded with curcumin (ALN-oHA-S-S-CUR) was formulated by conjugating the hydrophobic curcumin (a naturally derived compound) to hydrophilic oligosaccharides hyaluronan (oHA) via disulphide bonds. oHA is a hydrophilic polysaccharide that can selectively bind to the CD44 receptor, which is overexpressed in different tumour cells. In vitro, ALN-oHA-S-S-CUR showed higher uptake and cytotoxicity in MDA-MB-231 cells than in MCF-7 cells because the latter cell line has a lower expression of CD44 receptor. In a spheroid model mimicking the tumour in vivo, ALN-oHA-S-S-CUR showed a deeper penetration in the multicellular 3D MDA-MB-231 cell spheroid than in the non-targeted group. [95]. The researchers subsequently tested ALN-oHA-S-S-CUR in vivo, and it showed high binding affinity, robust antitumour, and anti-resorption activity in MDA-MB-231-burdened mice [96].

#### 5.1.4. Bortezomib and Curcumin

Alendronate-targeted nanoparticles composed of poly (D, L-lactide-co-glycolic) acid (PLGA) loaded with curcumin and bortezomib were designed by Thamake and co-workers. In vitro bone targeting studies indicated that these targeted nanoparticles had increased affinity to the bone tissues. Furthermore, the alendronate conjugated nanoparticles could localise at a significantly higher rate and quantities with a more prolonged accumulation at the tumour site than the control groups in a bone metastasis model induced by intra-tibia injection of MDA-MB-231 model in vivo. Furthermore, the targeted nanoparticles could inhibit the process of bone resorption and tumour growth in vivo. However, the combination of curcumin and bortezomib did not exert any synergistic effect in the anti-osteoclastogenic activities in the bone [97].

#### 5.1.5. Doxorubicin

Zhao and co-workers designed a pH, and redox dual sensitive core crosslinked nanoparticle (DOX@ALN-(HA-PASP)_CL_) loaded with doxorubicin. These nanoparticles were based on hyaluronic acid (HA) for specific binding to CD44 receptors expressed in BC cancer cells, and poly (aspartic acid) (PASP) DOX@ALN-(HA-PASP)_CL_ showed enhanced bone affinity compared to the non-targeted nanoparticles in in vitro and in vivo assays. In an anti-bone resorption study in in vitro 3D model of breast cancer bone metastasis, the number of bone lacunas and osteoclasts induced by MDA-MB-231 cells at the calvarial sites were significantly decreased in DOX@ALN-(HA-PASP)_CL_ -treated groups compared to the control groups, which indicated the anti-bone resorption ability of these two nanoparticles. Among MDA-MB-231 bearing mice, the administration of DOX@ALN-(HA-PASP)_CL_ could repress tumour growth and significantly increase the ratio of bone volume to tissue volume, which is an indicator of less bone resorption [98].

Pham et al. fabricated a doxorubicin-loaded, alendronate-modified DDS based on PLGA, which showed significant bone targeting, antitumour and anti-bone resorption [99]. A graphene oxide nanosheets (NGOs) doxorubicin-loaded DDS was developed with alendronate as the targeting ligand (NGO-AL). The in vivo biodistribution assay results indicated that NGO-ALs could localise more and show a longer retention time in the metastatic bone lesions compared to the non-targeted NGOs among MDA-MB-231 bearing BABL/c nude mice [100].

Wu et al. developed an alendronate and low molecular weight heparin functionalised liposome (A-L-DOX-Lip) to achieve the selective delivery of doxorubicin. The low molecular weight heparin could increase the blood circulation time and exert anti-metastasis activity. With the combination of alendronate and heparin, A-L-DOX-Lip could significantly reduce the tumour volume at bone metastatic sites in a bone metastasis model induced by intra-femoral injection of 4T1 cells, decrease the toxicity of doxorubicin, and reduce bone resorption [101].

Another alendronate-modified, doxorubicin-loaded, pH-sensitive DDS (ALN-PEG/C18/HYD-DOX-g-PASPAM) was reported by Lim et al., which could reduce the tumour volume in vivo in a mouse tumour model induced by subcutaneous injection of MDA-MB-231 cells compared to free doxorubicin and non-targeted particles [89].

#### 5.1.6. Docetaxel

An alendronate-functionalised amphiphilic triblock micelle based on PEG, polyglutamic acid, and polyphenylalanine (PEG-PGlu-PPhA) (ALN-m/DTX) was prepared to deliver docetaxel to metastatic sites of BC. In in vitro studies, ALN-m/DTX could exert a potent cytotoxic effect in a 4T1 cell model that was mimicking the bone microenvironment with hypercalcemia. Those micelles also showed an improved capability for binding to the bone and inhibiting the progress of osteoclastogenesis, bone resorption, and tumour-induced macrophage migration both in vitro and in vivo. In addition, the development of bone metastasis was delayed, and the weight loss was significantly controlled after the administration of ALN-m/DTX in a mouse BC bone metastasis model induced by injecting 4T1 cells into the left cardiac ventricle of female BALC/c mice [58].

Another amphophilic phospholipid polymer functionalised with alendronate (PMBA-DTX) was developed to carry docetaxel. The targeted nanoparticles showed antitumour activity among various BC cell lines (MDA-MB-231. MCF-7 and 4T1) in vitro and an enhanced accumulation at bone tissue in vivo [102].

PEG-ylated polybutyl cyanoacrylate (PBCA) based zoledronic acid decorated nanoparticles (PBCA-PEG-ZOL) were formulated by Monkkonen’s group as a carrier of docetaxel. PBCA-PEG-ZOL could significantly increase the number of apoptotic cells compared to the docetaxel control group among MDA-MB-231 and BO2 cell lines, indicating an improved cytotoxic activity of docetaxel in these nanoparticles [103].

Being a good target for treating BC bone metastasis, ανβ3 integrin can be targeted by ligands other than RGD peptide. A quinolone nonpeptide ligand specific for ανβ3 integrin was developed and conjugated to phospholipid/polysorbate 80 micelle nanoparticles (ανβ3-MPs) to deliver docetaxel-prodrug to metastatic bone sites. In vivo, ανβ3-MPs showed a 6.5-fold higher affinity for bone tissue compared to non-targeted MPs. Furthermore, after the administration of ανβ3-MPs/DTX-PD, the tumour volume and tumour-induced bone loss were significantly reduced with less hepatotoxicity compared to the docetaxel-treated group [104].

#### 5.1.7. Paclitaxel

A PLGA-based DDS with two targeting agents was designed, alendronate (A) for bone targeting and folic acid (FA) for targeting cancer cells that overexpress the folate receptors. Paclitaxel was loaded in the hydrophobic pocket of D-α-tocopheryl polyethylene glycol succinate (TPGS), and different targeted NPs were fabricated. Considering the HA binding test results, in vitro cellular up-taking test, and in vitro cytotoxicity test, the PTX-AFTPNs (A to F ratio: 0.67) showed the best dual-targeting potency and was further studied in vivo. Although both mono (A) and dual (AF) targeted NPs, i.e., ATPNs and AFTPNs (A:F = 0.67), were localised in bone lesions more than the control groups, mono-targeting nanoparticles (ATPNs) exhibited higher affinity to the bone than AFTPNs (A:F = 0.67). This effect was probably due to the presence of FA that decreases the percentage of alendronate on the surface of nanoparticles. PTX-AFTPNs showed the highest antitumour activity at the metastatic bone sites of 4T1 tumour-bearing mice, the best survival rate and an improved bone morphological integrity [88].

A novel alendronate-targeted paclitaxel-loaded pullulan (Pull) delivery system (Pull-(GGPNle-φ-PTX)-(PEG-ALN)) developed and showed robust affinity to bone tissue in vitro. In addition, these nanoparticles could also inhibit the proliferation of BC cells (MDA-MB-231 and 4T1) and suppress BC cell migration and angiogenesis [105].

In another study, a glutamic oligopeptides-RGD peptide derivative (Glu6-RGD) was designed as a targeting agent for conjugation to paclitaxel-loaded liposomes to enhance the biodistribution to the bone in BC bone metastasis. In vivo studies showed that the paclitaxel concentration in the bone metastatic lesions of targeted liposome treated mice was significantly higher than in free paclitaxel treated mice. Moreover, the liposome accumulated significantly more in metastatic bone lesions compared to normal bones [106].

#### 5.1.8. Non-Cytotoxic Payloads

Apart from the traditional anticancer compounds, alendronate-functionalised nanoparticles could also be an effective carrier for specific inhibitors of transcription factors. A small molecular inhibitor of transcription factor Gli2 (an upregulator of PTHrP), GANT58, was encapsulated in alendronate-conjugated amphiphilic diblock copolymer-based nanoparticles (GANT58-BTNPs). It was reported that 10% of alendronate (based on molar ratios) in the hydrophilic polymer block gave rise to the best balance between systemic pharmacokinetics and bone affinity. This amount of alendronate resulted in the highest bone to live biodistribution ratio. The administration of GANT58-BTNPs could significantly inhibit tumorigenesis and bone resorption in a mouse intracardiac model of bone metastasis. The bone volume fraction in the tibiae of the GANT58-BTNPs treated group was significantly higher than drug-free alendronate-conjugated nanoparticles, indicating that alendronate and GANT58 could exert dual beneficial therapeutic effects [107].

### 5.2. Targeted DDSs with Immunostimulatory Payloads

Bisphosphonates such as zoledronic acid have also been used for the selective delivery of immunostimulatory agents. Pang and co-worker designed zoledronic acid-modified bone targeting metal-organic framework (MOF) nanoparticles loaded with immunostimulatory cytosine-phosphate-guanosine (CpG) (BT-isMOF). Both results from in vitro and in vivo studies indicated that BT-isMOF nanoparticles had a robust capability of targeting the metastatic bone lesions, leading to a significant reduction in the osteoclast-mediated bone resorption and simultaneous induction of macrophage polarisation to the M1 pro-inflammatory phenotype. This phenotype is known for the secretion of pro-inflammatory cytokines, which may also play a role in the antitumour activities [108].

### 5.3. Targeted DDSs Loaded with Contrast Agents

Bisphosphonate-conjugated nanoparticles loaded with contrast agents could play a role in diagnosing BC bone metastasis. Qiao et al. developed zoledronic acid-conjugated gadolinium (Ⅲ) upconversion nanoparticles (PUCZP) by encapsulating plumbagin and poly (acrylic acid) (PAA) inside bimodal mesoporous silica. With the existence of gadolinium (Ⅲ), a contrast agent in T1-MRI, PUCZP could help to detect early bone metastasis, which is generally hard to diagnose by standard radiography. With the help of PAA, the nanoparticles could release in a pH-sensitive mode in the osteoclast acidity (pH = 4.5~5.5). Furthermore, PUCZP could inhibit the expression of RANKL and further suppress the osteocyte-induced osteoclast formation and weaken the invasive properties of MDA-MB-231 and 4T1 cells in vitro. In addition, UPCZP could repress tumour growth and osteoclastogenesis in a mouse intracardiac model of BC bone metastasis [90].

### 5.4. Targeted DDSs Loaded with Photothermal Therapeutic Agents

Photothermal therapy (PTT) is a method that converts absorbed photon energy to heat by utilising photo-absorbing materials and killing the cancer cells under a near-infrared (NIR) laser. According to relevant studies, PTT is capable of antitumour activity by itself and could increase the sensitivity of tumour cells to anticancer compounds, which further improves the efficacy of chemotherapy [109]. In addition, PTT shows favourably non-invasive and controllable features. If combining the antitumour drugs with photothermal agents, the NIR laser could be regarded as a trigger to promote the release of the loaded drugs [110]. Zoledronic acid is widely employed in those nanoparticles designed as carriers for photothermal agents.

Nanoparticles conjugated with zoledronic acid were designed by encapsulating gold nanorods in mesoporous silica (Au@MSNs-ZOL). The Au@MSNs-ZOL showed great affinity for bone both in vitro and in vivo. In in vitro studies, the Au@MSNs-ZOL could promote the differentiation of osteoblasts and inhibit the formation of osteoclast-like cells. Furthermore, combining with NIR, Au@MSNs-ZOL could significantly reduce the volume and weight of the tumour among MDA-MB-231 bearing mice, which could relieve bone pain and reduce the bone resorption at the metastatic bone lesions [59].

Superparamagnetic iron oxide (Fe_3_O_4_) and indocyanine green (ICG)-loaded PLGA nanoparticles (ICG/Fe_3_O_4_@PLGA-ZOL) were prepared, on which zoledronic acid was conjugated as a targeting agent. In vitro, the group with magnet and laser showed less cell viability than the group without a magnet at the bottom of the plate. More nanoparticles were engulfed by the cells and killed under irradiation. In this DDS, both zoledronic acid and Fe_3_O_4_ play the targeting role, which results in a high affinity to bone and great anti-tumorigenic potency [111].

### 5.5. Targeted DDSs Loaded with Photodynamic Therapeutic Agents

Photodynamic therapy (PDT) is a non-invasive, safe, and selective therapeutic approach widely studied in treating various kinds of cancers. With a combination of different factors, including a photosensitiser, an appropriate wavelength, and molecular oxygen, PDT could generate reactive oxygen species (ROS), leading to cell necrosis or apoptosis [112,113]. Significantly, only cells with intracellular photosensitiser will be damaged, ensuring selectivity and safety [113].

An alendronate-functionalised DDS (BTZ@ZnPc-ALN) was developed for the selective co-delivery of bortezomib and a photosensitiser Zinc phthalocyanine to achieve the chemo-PDT of BC bone metastasis. In vivo studies showed that BTZ@ZnPc-ALN could reduce the tumour volume by 85% compared to the control group in MDA-MB-231 bearing mice, which was realised by inducing ROS-induced mitochondrial damage. In addition, the expression of GRP78 protein and the cytosolic Ca^2+^ levels increased, resulting in excessive endoplasmic reticulum stress leading to the inhibition of tumour cell proliferation [91]. Detailed information about targeted DDSs for prevention and treatment of BC bone metastasis is summarised in Table 3.

## 6. Conclusions and Perspectives

As the most common site of metastasis, bone metastasis seriously affects BC patients’ survival rate and quality of life and remains a challenging clinical condition. Therefore, we need effective treatments to avoid the devastating impacts of BC bone metastasis on patients. One way to achieve this could be the development of targeted DDSs. Nanotechnology-based delivery systems have shown several advantages in treating BC bone metastasis compared to the conventional therapies, including: (1) solve the challenges associated with poor physicochemical properties of the payloads such as low solubility of hydrophobic drugs; (2) increase the therapeutic index of drugs; (3) enhance the metabolic stability and plasma circulation times of the payloads; (4) promote passive targeting through the EPR effect and mediate active targeting to the bone using different targeting moieties; (5) enable triggered release of the payloads. With the conjugation of a targeting agent, these DDSs can specifically deliver payloads to the bone metastatic lesions, which can greatly reduce the side effect of the cytotoxic anticancer reagents. This review highlighted the research involving DDSs relevant to the targeted treatment of BC bone metastasis, suggesting promising therapeutic options for this unmet condition. However, being in their early preclinical stages of development, there are still challenges for the future translation of these technologies to the clinic. Some of those include optimising the fabrication process for upscaling to achieve clinical translation and designing and conducting studies that inform about the fate of DDSs in vivo and their interaction with blood, healthy and diseased tissues, and cells, as well as intracellular compartments. Furthermore, lessons from previous drug developments in the area of nanomedicine have shown that those DDSs with more complex designs face additional challenges in their optimisation and characterisation that leads to lower reproducibility in the production processes. To fully uncover the potential of nanotechnology-based DDSs in BC bone metastasis, it is necessary to understand the nanomaterials’ properties and the metastasis itself. This allows more in-depth investigations on the interaction between these DDSs and the BC bone metastasis.

## Figures and Tables

**Table 1:** Conventional treatments for BC bone metastasis [55].

Therapeutic Options	Main Indications
Systemic endocrine therapy	Disease control
Systemic chemotherapy	
Systemic targeted therapy	
Adjuvant bone-targeted therapy (bisphosphonates, denosumab)	SREs, bone loss and metastasis prevention
Radiotherapy	Bone pain relief Bone recalcification Metastatic spinal cord compression control (administered with concomitant steroids)
Surgical intervention	Bone pain relief Independence/mobility improvement SREs prevention
Analgesics	Chronic pain relief

**Table 2:** Differences among three generations of bisphosphonates.

Bisphosphonates	Generation	Name	R1	R2
Non-Nitrogenous	First	Etidronate	-OH	-CH3
	Second	Clodronate	-Cl	-Cl
		Tiludronate	-H	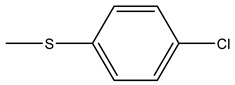
Nitrogenous		Pamidronate	-OH	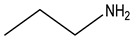
	Third	Alendronate	-OH	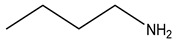
		Neridronate	-OH	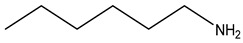
		Olpadronate	-OH	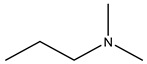
		Ibandronate	-OH	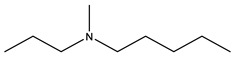
		Risedronate	-OH	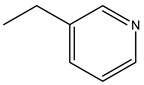
		Zoledronic acid	-OH	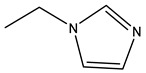

**Table 3:** A summary of targeted therapeutics for BC bone metastasis.

Nanoparticle	Particle Size (nm)	Particle Type	Zeta Potential (mV)	Targeting Agent	Loaded Compound
Zn@PEG-ALN NPs	About 55 *	Polymeric nanoparticle	About −25 *	Alendronate	Cisplatin prodrug
DZ@ALN	61 ± 0.78	Polymeric nanoparticle	−23.5 ± 0.41	Alendronate	Cisplatin prodrug and Zoledronate
ALN-NPs	95 ± 15	Micelle	−11.7 ± 4.3	Alendronate	Bortezomib
ALN-oHA-S-S-CUR	179 ± 23	Micelle	−25.7 ± 0.7	Alendronate	Curcumin
ALN-oHA-S-S-CUR	180	Micelle	/	Alendronate	Curcumin
Alendronate coated PLGA nanoparticles	235.5 ± 71.3	Polymeric nanoparticle	/	Alendronate	Bortezomib and Curcumin
DOX@ALN-(HA-PASP)_CL_	110 ± 9	Polymeric nanoparticle	−16.3 ± 3.7	Alendronate	Doxorubicin
NGO-ALs	60–150	Nanosheet	/	Alendronate	Doxorubicin
A_1_-L-DOX-Lip A_10_-L-DOX-Lip	107.2 ± 4.8 106.5 ± 3.5	Liposome	−11.5 ± 1.96 −12.3 ± 2.01	Alendronate	Doxorubicin
ALN-PEG/C_18_/HYD-DOX-g-PASPAM	About 200	Micelle	/	Alendronate	Doxorubicin
ALN-m/DTX	84 ± 5	Micelle	−30 ± 2	Alendronate	Docetaxel
PMBA-DTX	27.0 ± 0.1	Micelle	−11.8 ± 1.6	Alendronate	Docetaxel
PTX-AFTPNs (A to F ratio: 0.67)	125.9 ± 0.95	Polymeric nanoparticle	−29.6 ± 1.21	Alendronate	Paclitaxel
Pull-(GGPNle-φ-PTX)-(PEG-ALN)	163.3 ± 18.3 (pH = 5.5)	Micelle	/	Alendronate	Paclitaxel
GANT58-BTNPs	About 100	Micelle	/	Alendronate	Small molecule inhibitors of Gli2
BTZ@ZnPc-ALN	About 60	Polymeric nanoparticle	−18 mV	Alendronate	Bortezomib and Zinc phthalocyanine
Au@MSNs-ZOL	About 70	Mesoporous silica nanoparticle	+24.3	Zoledronic acid	Gold nanorods
BT-isMOF	228 ± 12	Metal−organic framework nanoparticle	/	Zoledronic acid	Immunostimulatory oligonucleotide
PBCA-PEG-ZOL NPs	82 ± 6.35	Polymeric nanoparticle	From −8.26 ± 1.26 to −23.51 ± 3.37	Zoledronic acid	Docetaxel
UCZP	About 60	Mesoporous silica nanoparticle	−18.9	Zoledronic acid	Gadolinium
ICG/Fe_3_O_4_@PLGA-ZOL	313.9	Polymeric nanoparticle	−15.0	Zoledronic acid	Iron oxide (Fe_3_O_4_) and indocyanine green
DPA−G5-PEG−cRGD/BTZ	78.02 *	Polymeric nanoparticle	−3.425 *	RGD peptide	Bortezomib
PTX-Glu6-RGD-Lip	121.9 ± 4.7	Liposome	−14.37 ± 4.85	RGD peptide (Glu6-RGD) derivative	Paclitaxel
αvβ3-MPs	12.5 ± 0.8	Micelle	−3.82 ± 1.23	Quinolone nonpeptide	Docetaxel

* The exact value was not given in the original paper; the value is obtained from the size distribution graph by the author of this article.
